# Serious Safety Signals and Prediction Features Following COVID-19 mRNA Vaccines Using the Vaccine Adverse Event Reporting System

**DOI:** 10.3390/ph17030356

**Published:** 2024-03-10

**Authors:** Jung Yoon Choi, Yongjoon Lee, Nam Gi Park, Mi Sung Kim, Sandy Jeong Rhie

**Affiliations:** 1 Graduate School of Pharmaceutical Sciences, Ewha Womans University, Seoul 03760, Republic of Korea; jungyoonchoi@ewhain.net (J.Y.C.); ngpark93@ewhain.net (N.G.P.); miseong@ewhain.net (M.S.K.); 2 Conpapa Inc., Seoul 06164, Republic of Korea; ryan@conpapa.co

**Keywords:** serious adverse reactions, signal detection, prediction model, mRNA-based COVID-19 vaccine, VAERS

## Abstract

We aimed to analyze the characteristics of serious adverse events following immunizations (AEFIs) to identify potential safety information and prediction features. We screened the individual case safety reports (ICSRs) in adults who received mRNA-based COVID-19 vaccines using the Vaccine Adverse Event Reporting System until December 2021. We identified the demographic and clinical characteristics of ICSRs and performed signal detection. We developed prediction models for serious AEFIs and identified the prognostic features using logistic regression. Serious ICSRs and serious AEFIs were 51,498 and 271,444, respectively. Hypertension was the most common comorbidity (22%). Signal detection indicated that the reporting odds ratio of acute myocardial infarction (AMI) was more than 10 times. Those who had experienced myocardial infarction (MI) were 5.7 times more likely to suffer from MI as an AEFI (95% CI 5.28–6.71). Moreover, patients who had atrial fibrillation (AF), acute kidney injury (AKI), cardiovascular accident (CVA), or pulmonary embolism (PE) were 7.02 times, 39.09 times, 6.03 times, or 3.97 times more likely to suffer from each AEFI, respectively. Our study suggests that vaccine recipients who had experienced MI, AF, AKI, CVA, or PE could require further evaluation and careful monitoring to prevent those serious AEFIs.

## 1. Introduction

In December 2019, the severe acute respiratory syndrome coronavirus 2 (SARS-CoV-2) first emerged and on 11 March 2020, it was declared a pandemic [1]. The World Health Organization (WHO) named the resultant disease complex coronavirus disease 2019 (COVID-19) [2]. Clinical consequences varied from asymptomatic cases to severe acute respiratory distress syndrome (ARDS) and death. According to data provided by the United States (U.S.) Centers for Disease Control and Prevention (CDC), as of 27 April 2022, more than 80 million people had been infected with SARS-CoV-2 in the U.S. and 980,000 of them had died. A vaccine can induce protective antiviral reactions against SARS-CoV-2, making it the most effective way, aside from containment, to hinder infection in vulnerable individuals [2]. In the context of a pandemic, the U.S. Food and Drug Administration (FDA) authorized the emergency use of two mRNA vaccines against SARS-CoV-2, which were the Pfizer–BioNTech vaccine (BNT162b2) and the Moderna vaccine (mRNA-1273) on 11 December 2020, and 18 December 2020, respectively. Since their approval, approximately 80% of the American population has received at least one dose of the COVID-19 vaccine [3].

However, there are concerns regarding the adverse events (AEs) after administrating the vaccine because these are vector-based vaccines, which were developed through a new mechanism using a new platform, mRNA, and were subjected to a limited time of post-vaccination follow-up due to their fast-track approval.

Vaccines are designed to be effective and safe, yet undetected adverse events following immunizations (AEFIs) can occur in post-market clinical trials, and there have been reports regarding the substantial number of AEFIs [4,5,6]. Particularly, within the special situation of a pandemic, when serious AEFIs such as death are reported by the media, the public’s trust in vaccines decreases due to fear of potential AEFIs, which might lead to the rejection of, or hesitancy in, vaccines [4,5].

It is important to evaluate the incidence and risk factors of AEFIs, especially for clinically serious AEFIs. Currently, there are studies on the commonly reported AEFIs; however, a limited number of studies have analyzed the serious AEFIs [7,8]. In a 2021 survey of scientists in Nature [9], 90% agreed that SARS-CoV-2 will become an endemic virus along with the therapeutic strategies developed for symptoms [10], meaning that the vaccine is expected to be continuously administered. In order for future vaccinations to be successful and safe, it is important to obtain information, especially on serious AEFIs. Ultimately, this will promote individual health and reduce the future burden of COVID-19.

The purpose of this study was to evaluate demographic characteristics and the prevalence of frequently reported AEFIs, collected in both serious and non-serious individual case safety reports (ICSRs) using vaccination [11]. More importantly, serious adverse events (SAEs) after the vaccination, which had not yet been identified in the authorization information, were investigated through signal detection using data mining methods [12]. Furthermore, we attempted to develop prediction models of the occurrence of serious AEFIs that could apply the features for monitoring and preventing the AEFIs in individuals after immunization.

## 2. Results

### 2.1. ICSRs by Demographic Characteristics

A total of 555,033 ICSRs were obtained for adults who received either of the COVID-19 mRNA vaccines. (Table 1). Moreover, the incidence of serious ICSRs was 28,267 (10.61%) for the Pfizer–BioNTech vaccine (BNT162b2) and 23,231 (8.05%) for the Moderna vaccine (mRNA-1273). Among ICSRs collected for the Pfizer–BioNTech vaccine (BNT162b2), 7.91% were for hospitalization/prolonged and 1.47% were for death. In relation to the Moderna vaccine (mRNA-1273), 5.93% were for hospitalization/prolonged and 1.33% were related to death (Table S3).

The serious ICSRs were higher among females for both mRNA-based COVID-19 vaccines (54.4% in the Pfizer–BioNTech vaccine (BNT162b2), 52.2% in the Moderna vaccine (mRNA-1273), *p* < 0.0000). Moreover, the subjects with serious ICSRs were significantly older than those with non-serious ICSRs (60.9 ± 19.0 vs. 48.4 ± 16.7, *p* < 0.0000 for the Pfizer–BioNTech vaccine (BNT162b2), 63.0 ± 18.5 vs. 52.5 ± 17.5, *p* < 0.0000 for the Moderna vaccine (mRNA-1273)). The incidence of serious ICSRs was significantly higher than those of non-serious ICSRs at 65 years old and above (Table 1).

The incidence rates of serious ICSRs were significantly higher than those of non-serious ICSRs when the time to onset of AEFIs exceeded 7 days (*p* < 0.0000), when more visits to the emergency or urgent care wards (*p* < 0.0000), more visits to offices or clinics (*p* < 0.0000), less recovery from the AEFIs (*p* < 0.0000), and more for the presence of the top five comorbidities (*p* < 0.0000). Hypertension was the most common disease among the individuals who reported serious ICSRs (*n* = 6247, 22.1%), followed by type 2 diabetes (12.3%), and hyperlipidemia (10.7%) for the Pfizer–BioNTech vaccine (BNT162b2) (Table 1).

### 2.2. Serious AEFIs

The total number of the serious AEFIs was 271,144, which included 150,575 (14.8%) from the Pfizer–BioNTech vaccine (BNT162b2) and 120,869 (10.6%) from the Moderna vaccine (mRNA-1273) (Table S3). The frequently reported serious AEFIs, classified by the system organ class (SOC) and preferred term (PT), are shown in Table 2.

The most frequently reported serious AEFIs were the ‘General disorders and administration site conditions’ in SOC term (21.7% from the Pfizer–BioNTech vaccine (BNT162b2) and 23.54% from the Moderna vaccine (mRNA-1273)). The PTs of this SOC included death, pyrexia, and fatigue. The SOC of serious AEFIs related to ‘nervous system disorders’ closely followed, with 14.5% reporting them after the Pfizer–BioNTech vaccine (BNT162b2) and 14.9% after the Moderna vaccine (mRNA-1273), while the respective AEFIs were headache, dizziness, and cerebrovascular accident (CVA) (0.8% in total). Finally, the diagnoses with high severity such as pulmonary embolisms (PE) (0.9% in total) were also noted (Table 2).

### 2.3. Disproportionality Analysis for Signal Detection of Serious AEFIs

The 201 and 110 signals satisfied the criteria for the proportional reporting ratio (PRR), reporting odd ratio (ROR), and information component (IC) for the Pfizer–BioNTech (BNT162b2) and Moderna (mRNA-1273) vaccines for all the reported AEFIs (Tables S5 and S6). Importantly, the signals for the serious AEFIs totaled 28 for the Pfizer–BioNTech vaccine (BNT162b2) and 37 for the Moderna vaccine (mRNA-1273) (Tables S7 and S8).

We identified three SOCs (‘cardiac disorders’, ‘infections and infestations’, and ‘renal and urinary disorders’) that increased the percentage change of the serious AEFIs compared to non-serious AEFIs by more than 200% (Table S8). Individually, the ROR for ‘cardiac disorders’ was more than three times higher after the Pfizer–BioNTech vaccine (BNT162b2) (ROR [95% CI]: 3.12 [2.91–3.34]) and Moderna vaccine (mRNA-1273) (3.24 [3.02–3.48]), than after the other vaccines. Similarly, the RORs for ‘infections and infestations’ and ‘renal and urinary disorders’ were more than twice as high with the Pfizer–BioNTech vaccine (BNT162b2) (‘infections and infestations’ 2.62 [2.50–2.75], ‘renal and urinary disorders’ 2.18 [1.95–2.44]) and the Moderna vaccine (mRNA-1273) (‘infections and infestations’ 2.16 [2.06–2.27], ‘renal and urinary disorders 2.04 [1.82–2.88]) than with all the other vaccines (Table 3).

Based on the PT, high RORs were presented for acute myocardial infarction (AMI) and appendicitis, which were illustrated to be more than 10 times higher than in the other vaccines (AMI: 10.75 [6.20–18.66] and appendicitis: 17.65 [7.88–39.56] for the Pfizer–BioNTech vaccine (BNT162b2); AMI: 10.27 [5.90–17.86] and appendicitis: 10.36 [4.59–23.40] for the Moderna vaccine (mRNA-1273)). Those signals were closely followed by acute kidney injury (AKI) and PE for each vaccine (AKI: 7.51 [5.48–10.31] and PE: 8.27 [6.14–11.13] for the Pfizer–BioNTech vaccine (BNT162b2); AKI: 5.79 [4.20–7.97] and PE: 9.66 [7.17–13.02] for the Moderna vaccine (mRNA-1273)). Additionally, the signals for atrial fibrillation (AF) and CVA were also detected for serious AEFIs (Table 4).

### 2.4. Predicting the Incidence of the Serious AEFIs and the Associated Features

Algorithms were developed for predicting the incidence of the major signals of the serious AEFIs, such as myocardial infarction (MI) (including AMI), AF, AKI, CVA, and PE. The areas under the receiver operating characteristic curves (AUROCs) of all the algorithms were greater than 75% (AUROCs of the algorithms containing 20 features: MI: 76%, AF: 78%, AKI: 85%, CVA: 76%, and PE: 78%) (Table S9).

**Figure 1:**
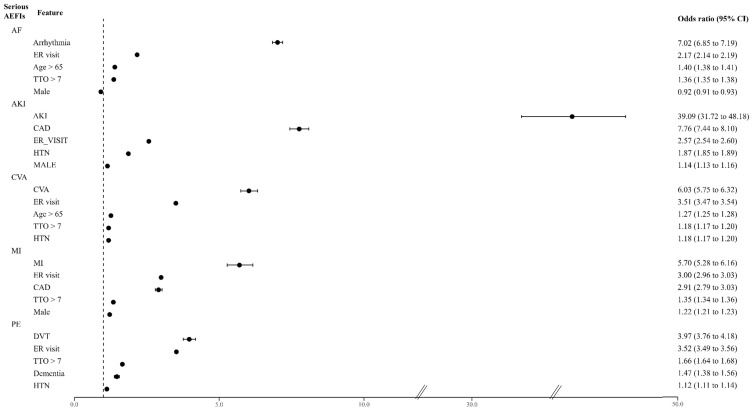
Major signals of the serious AEFIs and the associated predictors. AF: atrial fibrillation; AKI: acute kidney injury; CVA: cerebrovascular accident; MI: myocardial infarction (including acute MI); PE: pulmonary embolism; ER: emergency room; TTO: time to onset; CKD: chronic kidney disease; HTN: hypertension; CAD: coronary artery disease; DVT: deep vein thrombosis.

Among the numerous variables, five features were identified as highly dependent features through the recursive feature elimination (RFE) process used to predict the risk levels of each serious AEFI. For example, if a patient had an underlying disease of arrhythmia, the patient would have a 7.02 times higher risk of experiencing AF as an adverse reaction after receiving an mRNA-based COVID-19 vaccine (Figure 1). Interestingly, the features of the emergency room or urgent care visit (ER visit) and time to the onset (TTO) of AEFIs > 7 days were common features that increased the risk of all serious AEFIs (ER visit: 2.17 [2.14–2.19] for AF, 2.57 [2.54–2.6] for AKI, 3.51 [3.47–3.54] for CVA, 3.00 [2.96–3.03] for MI, and 3.52 [3.49–3.56] for PE; TTO of AEFIs > 7 days: 1.36 [1.35–1.38] for AF, 1.87 [1.85–1.89] for AKI, 1.18 [1.17–1.2] for CVA, 1.35 [1.34–1.36] for AMI, and 1.66 [1.64–1.68] for PE). Additionally, being over 65 years of age was a significant predictor associated with the serious signals of AF (1.40 [1.38–1.41]), AKI (1.14 [1.13–1.16]), and CVA (1.27 [1.25–1.28]). Sex also played a role, whereby males were associated with MI (1.22 [1.21–1.23]), and females were associated with AF (0.92 [0.91–0.93]) (Figure 1).

## 3. Discussion

We conducted an extensive investigation of a comprehensive dataset from the Vaccine Adverse Event Reporting System (VAERS) that included individual cases of safety from COVID-19 vaccine recipients and identified signals of SAEs through disproportionality analysis after the administration of two mRNA-based COVID-19 vaccines. We also utilized a machine learning-based regression approach to robustly predict the qualitative and quantitative SAEs (such as AMI) in the study by establishing associations with demographic features such as comorbidity and ER visits.

As the COVID-19 pandemic has progressed to the current endemic phase where long COVID and other exacerbating syndromes exist, vaccination has become inevitable. However, concerns regarding AEs from these vaccines have quickly emerged, despite many studies reporting on the development and effectiveness of various vaccines.

Vaccine safety studies have been conducted using healthcare vigilance data or spontaneous reporting systems for AEs, but many of them were carried out during the early stages of vaccine launch or for a short time period [13,14]. Moreover, the urgent demand for COVID-19 mRNA vaccines led to phased distribution strategies by the CDC’s Advisory Committee on Immunization Practices (ACIP) [15], which may have contributed to potential overestimation or missing of certain AEFIs. In addition, biased interpretation of AEFIs due to anxiety over COVID-19 complications and the influence of social media has also been a concern. Therefore, in order to obtain unbiased and meaningful outcomes on vaccine safety, both qualitative and quantitative aspects need to be considered, and large-scale and accumulated safety data are still required [16]. Our data were derived from a national surveillance data system, which includes AEFI cases reported by the public and groups of professionals at any time point from the initial stage to the stabilization stage since the time the vaccine was approved.

We found that there were statistical differences in reported cases by age and sex. Women reported higher rates of both serious and non-serious cases compared to men, which is consistent with previous findings [17,18,19]. However, a study analyzing AEs following administration of BNT162b2 to healthcare workers showed no sex differences in the frequency of AEFIs [20]. During a pandemic, non-serious cases may be underreported while the reporting of serious cases may increase, which could explain this difference [17]. Previous studies have also shown that age did not show a significant difference in the reporting of non-serious cases, but a significant increase was observed in the reporting of serious cases in individuals aged 65 and older, which is consistent with our results [21]. We also found a statistical difference between non-serious cases and serious cases in terms of onset intervals, with more than 50% of serious cases occurring after 7 days [22], indicating the need for close monitoring and long-term follow-ups for serious AEFIs.

The signals of cardiac disorders, infections and infestations, and renal and urinary disorders in the SOC were derived from individual case reports with terms such as acute myocardial infarction, atrial fibrillation, pulmonary embolism, acute kidney injury, and cerebrovascular accident from both vaccines. These manifestations are distinct from typical presentations occurring in patients with chronic cardiovascular and renal conditions and are likely to be sudden onset of acute events. Notably, these serious and acute AEFIs are not mentioned in the summary of product characteristics [23,24], suggesting the need for updates. Moreover, these serious AEFIs were classified as disorders in major organs according to PT classification and were attributed to major adverse cardiovascular events (MACE), while acute kidney injury is a known cause of major adverse kidney events (MAKE), including chronic kidney disease [25,26].

In the causal assessment of AEFIs, it was very important to differentiate other causes, especially the underlying diseases [27]. Although our study did not fully explain the mechanistic basis of vaccine-induced serious disorders, our models revealed that underlying comorbidity in vaccine recipients was a significant predictor of serious AEFIs in organs that share pathophysiological pathways with the disorders. Additionally, an age of 65 and older and ER visits were also predictors of serious adverse events, possibly due to the fragility and debilitating health conditions that may not be sufficient to overcome the antibody-producing reactions following immunization. Previous studies using medical records and national health data did not find statistically significant associations between COVID-19 vaccines and these serious AEFIs [28,29], while a recent study in Israel reported an increase of over 25% in emergency calls related to cardiac arrest and acute coronary syndrome in the age group of 16–39 during the COVID-19 vaccination rollout compared to the same period in previous years [30]. Another recent study showed that mRNA-based COVID-19 vaccines induced significant increases in inflammatory markers and decreased endothelial functions [31].

Our study had several limitations. Firstly, we did not consider the number of precedent vaccine doses in our analysis. While the number of doses could influence the occurrence of AEs, previous surveys in the U.S. and Vietnam have reported that the occurrences of AEs in adults were either similar or less frequent following booster vaccination [32,33]. Secondly, the data of the VAERS only reflect information on patients who experienced AEs following vaccination. Therefore, it may not conclusively establish the correlation between AEs and the vaccine, as it includes AEs that may have occurred merely in temporal association with the vaccine. Other factors, such as concomitant medications, and underlying diseases, are needed to elucidate the causal relationship. Nevertheless, our study identified an association between the frequency of individual AE reports of two COVID-19 vaccines and the vaccine-related characteristics of vaccine recipients such as time to onset of AEFIs, ER visits, prognosis, the top five comorbidities, and the frequency of various SAEs, which was aligned with findings from other studies [22]. Thirdly, the spontaneous reporting system inherently contains many omissions in the data, including information on underlying diseases, the number of doses, and dosage details. These omissions could potentially impact our logistic regression results. Fourthly, VAERS lacks data for comparable individuals who did not receive the vaccine, resulting in the absence of incidence rates in unvaccinated comparison groups. Therefore, the presented data consist solely of the number of individual case safety reports (ICSRs). Additionally, the variability in the quality of the reports needs to be carefully considered when interpreting the study results. Moreover, uncertainty regarding the diagnosis and suspicion of each AE at the time of reporting poses a challenge. Limitations with the use of data from self-reporting and surveillance systems include under- or over-reporting, simultaneous administration of multiple vaccine antigens, reporting bias, media interest, and the pandemic situation. Hence, a careful interpretation of results is warranted. Lastly, while the standard procedure involves developing a prediction model and conducting internal validation tests by splitting the data, external validation was not feasible due to the challenge of obtaining data similar to VAERS. Nevertheless, compared to studies commonly conducted to evaluate AEFIs, our study provided reliable evidence of vaccine safety through disproportionality analysis. Furthermore, VAERS data serve as a national post-marketing spontaneous reporting system for pharmacovigilance, functioning as an early warning system to detect safety issues. Our study analyzed a large dataset of over 500,000 case reports, reflecting high diversity. Additionally, the study period was sufficiently long after wide vaccine distribution with minimal restrictions on vaccine access and availability.

Despite these limitations, our study identified unspecified SAEs after the administration of mRNA-based COVID-19 vaccines using VAERS data. We also confirmed that individuals with comorbidities such as arrhythmia, coronary artery disease, thrombotic conditions, and renal conditions should be carefully monitored due to their higher potential for experiencing MI, AF, AKI, CVA, or PE after vaccination compared to those without underlying diseases. Further studies are needed to clarify the causality of these events and their potential association with the vaccine dose. This information can aid in identifying which adverse events should be labeled in the vaccine information and help pinpoint risk groups that may require close monitoring. Failure to do so could complicate the management of adverse events related to COVID-19 vaccines and contribute to increased vaccine hesitancy among the public.

## 4. Materials and Methods

### 4.1. Data Source

We used data from VAERS (https://vaers.hhs.gov (accessed on 3 February 2022)), which is currently considered the most intensive vaccine safety monitoring effort in the U.S. [34]. Three types of files were provided by VAERS. One was the VAERSDATA.csv file, which provided demographic information such as age, sex, the clinical status of their medical history, recovery status, and seriousness, the date of vaccine administration, the date of occurrence of AEFIs, etc. The second was a VAERSVAX.csv file, which contained vaccine information such as manufacturer, type, route, and dosing series. The third was a VAERSSYMPTOMS.csv file, which provided AEFI information with coded symptoms using the MedDRA (Medical Dictionary for Regulatory Activities) glossary. Each of the three files shared a VAERS ID in common [35]. Thus, we linked the VAERSVAX.csv file and the VAERSSYMPTOMS.csv file with the VAERS ID to create a new file, which matched the vaccines to the AEFIs with 1:1. Serious ICSRs refer to any of the included AEs as death, life-threatening, hospitalization or prolonged hospitalization, disability, and congenital anomaly/birth defect, which have previously been defined by the FDA and VAERS. We defined any AEFIs included in the serious ICSRs as ‘serious AEFIs’.

### 4.2. Patient Medical History Coding

The medical history of the patients was described in natural language after being obtained from the VAERS reporting form. Then, we classified the described medical conditions and medical abbreviations using MedDRA vocabulary for proper coding to perform the analysis (Table S2).

### 4.3. Study Design

#### 4.3.1. Incidences of ICSR and Serious AEFI

The reported ICSRs were collected for individuals 18 years and older, who had received their vaccine between 1 January 2017 and 31 December 2021. We excluded cases in which, (1) sex and age information were missing, (2) COVID-19 mRNA vaccines and other(s) were administered simultaneously (Figure S1). Each ICSR included one or more AEFIs. The AEFI data are provided by the MedDRA codes and terminology. The terminology is intended for use in recording AEs and medical history from pre-marketing to post-marketing, including diagnoses, signs and symptoms, investigations, etc. [36]. It consisted of five levels of system organ class (SOC), high-level group term, higher level term, preferred term (PT), and lowest level term. AEFI data were described using SOCs and PTs in this study (Figure S2). The protocol used in this study was exempt from review by the institutional review board of Ewha Womans University (ewha-202203-0029-01).

#### 4.3.2. Signal Detection for Serious AEFI

We performed signal detection for serious AEFI and all AEFIs twice based on the frequentist methods and Bayesian methods. The frequentist method includes proportional reporting ratio (PRR) and reporting odd ratio (ROR) [37,38], and the Bayesian method includes the multi-item Gamma Poisson shrinker (MGPS) and Bayesian confidence propagation neural network (BCPNN) (Table S1) [39,40]. We identified SOCs with a high reporting rate in serious AEFIs (at least 5000 cases) and calculated the RORs for these SOCs (Table S4).

#### 4.3.3. Prediction for Serious AEFIs

Prediction models for five serious AEFIs were developed using logistic regression models to calculate the odds ratio (ORs) with 95% confidence interval (CI)s. There were 41 types of variables available for those who received the 2 mRNA-based COVID-19 vaccines in the VAERS reports, which included information on 2 demographics (age, sex), time to the onset (TTO) of AEFIs, emergency room or urgent care visit, doctor or other healthcare provider office/clinic visit, and 36 comorbidities (Table S2).

We used data normalization and oversampling methods while preprocessing the data to handle the highly imbalanced data classifications and overcome the negligibility of the minority class [41]. Moreover, we used Recursive Feature Elimination (RFE) methods for feature selection using backward elimination by taking the given models and iterating the process over increasingly smaller feature subsets, until the best model hypothesis was achieved [42]. We randomly split the data into training and testing groups with a 70:30 ratio for model validation. The performance of the models was evaluated using the area under the receiver operating characteristic curve (AUROC) and calibration plots of observed versus predicted AEs, indicating the model’s discrimination power.

### 4.4. Statistical Analysis

We used descriptive analysis to summarize demographic and clinical information. Python statistical software version 3.10 (Python Software Foundation, Beaverton, OR, USA) was used for data cleaning, data mining, prediction analysis, and statistical analysis. The significance level was set at 0.05.

## 5. Conclusions

We comprehensively analyzed serious AEFIs and detected safety signals associated with two mRNA-based COVID-19 vaccines, Pfizer–BioNTech (BNT162b2) and Moderna (mRNA-1273), using data from VAERS. Furthermore, the study utilized machine learning-based regression models to assess the features and predict potential high-risk groups among vaccine recipients for the occurrence of serious AEFIs. This research contributes information for understanding the safety profile of mRNA-based COVID-19 vaccines. The potential associations between comorbidities and serious AEFIs emphasize the importance of ongoing surveillance and risk assessment to guide vaccination strategies and public health interventions.

## Figures and Tables

**Table 1:** Demographic characteristics and ICSRs after receiving the mRNA-based COVID-19 vaccines (*n* = 555,033).

Characteristics		Pfizer–BioNTech Vaccine (BNT162b2) (*n* = 266,369)		Moderna Vaccine (mRNA-1273) (*n* = 288,664)	
		Serious ICSR (*n* = 28,267)	Non-Serious ICSR (*n* = 238,102)	Serious ICSR (*n* = 23,231)	Non-Serious ICSR (*n* = 265,433)
		*n* (%)	*n* (%)	*n* (%)	*n* (%)
Sex	Female	15,368 (54.4)	172,592 (72.5)	12,132 (52.2)	196,952 (74.2)
	Male	12,899 (45.6)	65,510 (27.5)	11,099 (47.8)	68,481 (25.8)
Age (years)	Average ± SD	60.9 ± 19.0	48.4 ± 16.7	63.0 ± 18.5	52.5 ± 17.5
	18–64	14,784 (52.3)	192,886 (81.0)	10,907 (47.0)	187,333 (70.6)
	over 65	13,483 (47.7)	45,216 (19.0)	12,324 (53.1)	78,100 (29.4)
Time to onset of AEFIs (days)	0–7	12,004 (42.5)	194,233 (81.6)	10,612 (45.7)	199,417 (75.1)
	>7	15,398 (54.5)	32,432 (13.6)	11,934 (51.4)	51,702 (19.5)
	Unknown	865 (3.1)	11,437 (4.8)	685 (3.0)	14,314 (5.4)
Emergency room or urgent care visit	Visit	12,024 (42.5)	29,810 (12.5)	9041 (38.9)	22,932 (8.6)
	No visit	16,243 (57.5)	208,292 (87.5)	14,190 (61.1)	242,501 (91.4)
Doctor or other healthcare provider office/clinic visit	Visit	8005 (28.3)	55,531 (23.3)	5915 (25.5)	50,206 (18.9)
	No visit	20,262 (71.7)	182,571 (76.7)	17,316 (74.5)	215,227 (81.1)
Prognosis after the AEFIs	Recovered	4734 (16.8)	79,115 (33.2)	4639 (20.0)	97,522 (36.7)
	Not recovered	14,290 (50.6)	94,345 (39.6)	11,226 (48.3)	90,091 (33.9)
	Unknown	9243 (32.7)	64,642 (27.2)	7366 (31.7)	77,820 (29.3)
Top 5 comorbidity *	Hypertension	6247 (22.1)	23,523 (9.9)	5215 (22.5)	24,228 (9.1)
	Type 2 diabetes mellitus	3470 (12.3)	10,763 (4.5)	2992 (12.9)	11,357 (4.3)
	Hyperlipidemia	3028 (10.7)	8912 (3.7)	2543 (11.0)	9485 (3.6)
	Asthma	1428 (5.1)	14,640 (6.2)	1068 (4.6)	13,541 (5.1)
	Hypothyroid disease	1339 (4.7)	8756 (3.7)	1206 (5.2)	8682 (3.3)
	Unknown or vague description	12,751 (45.1)	130,241 (54.7)	10,907 (47.0)	168,965 (63.7)

* A report may contain more than one comorbidity.

**Table 2:** Frequently reported serious AEFIs after mRNA-based COVID-19 vaccination.

SOC	PT	Serious AEFI (Total *n* = 271,444)		Pfizer–BioNTech Vaccine (BNT162b2) (*n* = 150,575)		Moderna Vaccine (mRNA-1273) (*n* = 120,869)	
		*n*	%	*n*	%	*n*	%
General disorders and administration site conditions	Death	7694	2.8	4021	2.7	3673	3.0
	Pyrexia	6536	2.4	3455	2.3	3081	2.6
	Fatigue	5668	2.1	3160	2.1	2508	2.1
	Asthenia	5183	1.9	2765	1.8	2418	2.0
	Pain	4336	1.6	2420	1.6	1916	1.6
	Chest pain	4333	1.6	2439	1.6	1894	1.6
	Condition aggravated	3927	1.5	2200	1.5	1727	1.4
	Chills	3168	1.2	1659	1.1	1509	1.3
	Malaise	2648	1.0	1375	0.9	1273	1.1
	Others	17,656	6.5	9205	6.1	8451	7.0
	Total	61,149	22.5	32,699	21.7	28,450	23.5
Nervous system disorders	Headache	4517	1.7	2463	1.6	2054	1.7
	Dizziness	3503	1.3	1943	1.3	1560	1.3
	Cerebrovascular accident	2230	0.8	1139	0.8	1091	0.9
	Hypoaesthesia	2156	0.8	1264	0.8	892	0.7
	Others	27,345	10.1	14,981	10.0	12,364	10.2
	Total	39,751	14.6	21,790	14.5	17,961	14.9
Respiratory, thoracic, and mediastinal disorders	Dyspnoea	10,752	4.0	6042	4.0	4710	3.9
	Cough	5039	1.9	3093	2.1	1946	1.6
	Pulmonary embolism	2396	0.9	1237	0.8	1159	1.0
	Hypoxia	2259	0.8	1280	0.9	979	0.8
	Others	18,195	6.7	10,457	6.9	7738	6.4
	Total	38,641	14.2	22,109	14.7	16,532	13.7
Infections and infestations	COVID-19	14,143	5.2	8628	5.7	5515	4.6
	COVID-19 pneumonia	2907	1.1	1831	1.2	1076	0.9
	Pneumonia	2065	0.8	1115	0.7	950	0.8
	Others	9189	3.4	5324	3.5	3865	3.2
	Total	28,304	10.4	16,898	11.2	11,406	9.4
Gastrointestinal disorders	Nausea	3960	1.5	2155	1.4	1805	1.5
	Vomiting	3096	1.1	1652	1.1	1444	1.2
	Diarrhea	2567	1.0	1493	1.0	1074	0.9
	Others	9278	3.4	5155	3.4	4123	3.4
	Total	18,901	7.0	10,455	6.9	8446	7.0
Musculoskeletal and connective tissue disorders	Pain in extremity	3047	1.1	1668	1.1	1379	1.1
	Arthralgia	2209	0.8	1239	0.8	970	0.8
	Others	11,886	4.4	6556	4.4	5330	4.4
	Total	17,142	6.3	9463	6.3	7679	6.4

AEFI: adverse events following immunization; SOC: system organ class; PT: preferred term.

**Table 3:** Signals of serious AEFIs in SOC by disproportionality analysis.

SOC	Pfizer–BioNTech Vaccine (BNT162b2) ROR [95% CI]	Moderna Vaccine (mRNA-1273) ROR [95% CI]
Cardiac disorders	3.12 [2.91–3.34]	3.24 [3.02–3.48]
Infections and infestations	2.62 [2.50–2.75]	2.16 [2.06–2.27]
Renal and urinary disorders	2.18 [1.95–2.44]	2.04 [1.82–2.28]

SOC: system organ class; ROR: reporting odd ratio; CI: confidence interval.

**Table 4:** Signals of serious AEFIs in PT by disproportionality analysis.

PT	Pfizer–BioNTech Vaccine (BNT162b2)		Moderna Vaccine (mRNA-1273)	
	*n*	ROR [95% CI]	*n*	ROR [95% CI]
Acute myocardial infarction	467	10.75 [6.20–18.66]	358	10.27 [5.90–17.86]
Pulmonary embolism	1237	8.27 [6.14–11.13]	1159	9.66 [7.17–13.02]
Acute kidney injury	1001	7.51 [5.48–10.31]	620	5.79 [4.20–7.97]
Myocardial infarction	526	3.84 [2.79–5.28]	500	4.55 [3.31–6.26]
Atrial fibrillation	814	3.54 [2.76–4.52]	703	3.81 [2.97–4.87]
Cerebrovascular accident	1139	3.49 [2.84–4.29]	1091	4.17 [3.39–5.13]

PT: preferred term; ROR: reporting odds ratio, CI: confidence interval.

## Data Availability

A framework for applying the logistic regression prediction model built using Python is available in the ‘EWHAMLLogistic’ repository, https://github.com/EWHAResearch/EWHAMLLogistic. VAERS data is accessible by downloading raw data in CSV files for import into a database, spreadsheet, or text editing program, or by using the CDC WONDER online search tool. Personal identifying information of individuals who received the vaccine(s) will not be disclosed to the public.

## Supplementary Materials

The following supporting information can be downloaded at: https://www.mdpi.com/article/10.3390/ph17030356/s1. Figure S1: Selection of individual case safety reports; Figure S2: Selection of adverse events following immunization; Table S1: Definition and criteria of disproportionality analysis for signal detection; Table S2: List of highly reported patient medical history in VAERS; Table S3: Clinical consequences of serious ICSRs after mRNA-based COVID-19 vaccination (*n* = 555,033); Table S4: Percentage changes between serious AEFIs and non-serious AEFIs by SOC after mRNA-based COVID-19 vaccination; Table S5: Signals detected from adverse events after administration of Pfizer–BioNTech (BNT162b2); Table S6: Signals detected in adverse events after administration of Moderna (mRNA-1273); Table S7: Signals detected from serious adverse events after administration of Pfizer–BioNTech (BNT162b2); Table S8: Signals detected from serious adverse events after administration of Moderna (mRNA-1273); Table S9: Area under the receiver operating characteristic curve (AUROC) with various numbers of selected features in major signals of AEFIs.
