# Supplementary material for: Serious Safety Signals and Prediction Features Following COVID-19 mRNA Vaccines Using the Vaccine Adverse Event Reporting System
Source: Pharmaceuticals (Basel). 2024 Mar 10;17(3):356. doi: 10.3390/ph17030356 (PMC10974993; doi:10.3390/ph17030356)
Supplement: Supplementary file 1 [file pharmaceuticals-17-00356-s001.zip › pharmaceuticals-2837264-supplementary.pdf]

Serious Safety Signals and Prediction Features Following COVID-19 mRNA Vaccines Using the Vaccine Adverse Event Reporting System

Supplementary Appendix

Table of contents

Figure S1. Selection of Individual case safety reports. 2

Figure S2. Selection of adverse events following immunization. 3

Table S1. Definition and criteria of disproportionality analysis for signal detection. 4

Table S2. List of highly reported patient medical history in VAERS. 5

Table S3. Clinical consequences of serious ICSRs after mRNA-based COVID-19 vaccination (n=555,033). 6

Table S4. Percentage changes between serious AEFIs and non-serious AEFIs by SOC after mRNA-based COVID-19 vaccination. 7

Table S5. Signals detected from adverse events after administration of Pfizer-BioNTech (BNT162b2). 8

Table S6. Signals detected in adverse events after administration of Moderna (mRNA-1273). 13

Table S7. Signals detected from serious adverse events after administration of Pfizer-BioNTech (BNT162b2). 16

Table S8. Signals detected from serious adverse events after administration of Moderna (mRNA-1273). 17

Table S9. Area under the receiver operating characteristic curve (AUROC) with various numbers of selected features in major signals of AEFIs. 18

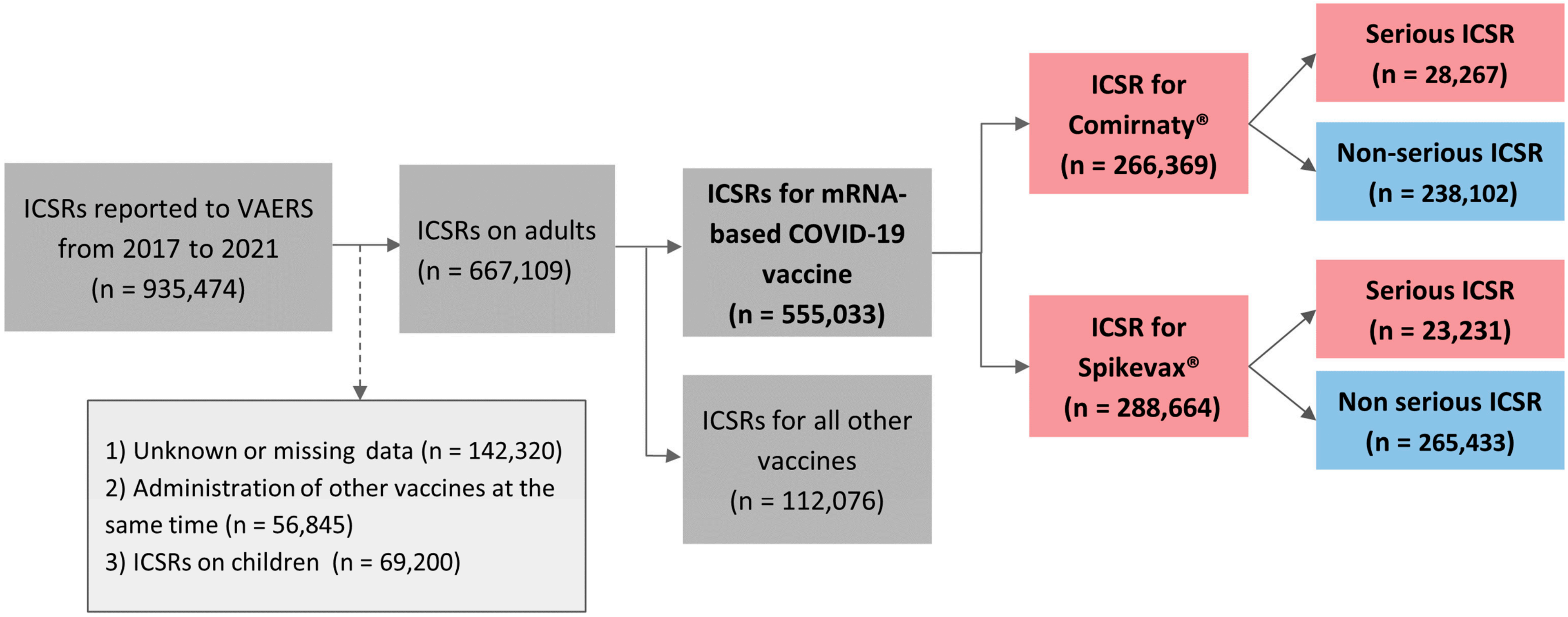

Figure S1. Selection of Individual case safety reports.

COVID-19: corona virus infectious disease-19; ICSR: individual case safety report; VAERS: vaccine adverse event reporting system

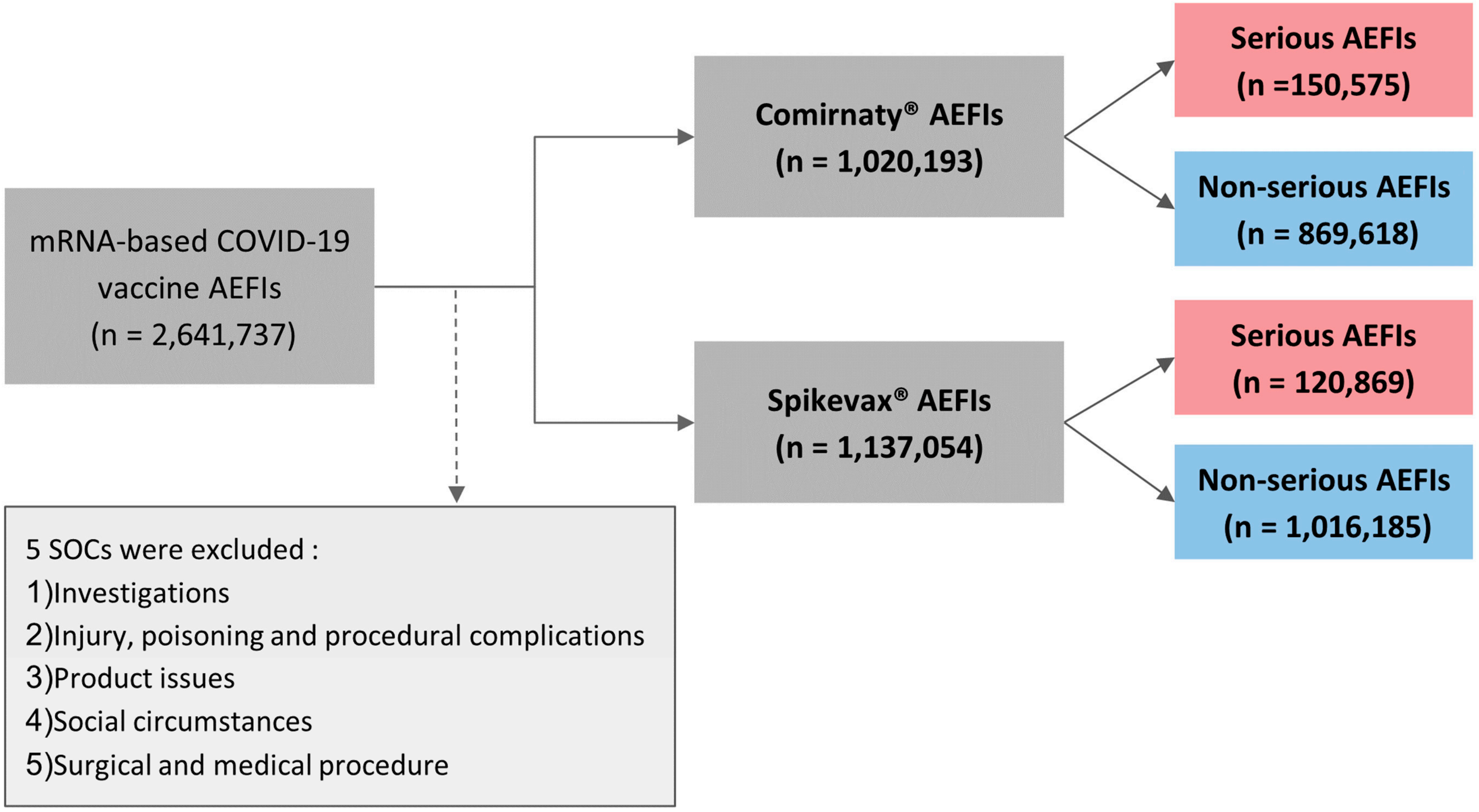

Figure S2. Selection of adverse events following immunization.

AEFI: adverse events after immunization; COVID-19: corona virus infectious disease-19; SOC: system organ class

Table S1. Definition and criteria of disproportionality analysis for signal detection.

Number of reports	A specific AE	All other AEs
**Target mRNA vaccine**	A	B
**All other vaccines**	C	D

AE = adverse event

Measures	Calculation	Criteria as signal
PRR	PRR= (A / (A + B)) / (C / (C + D))	PRR ⩾2, χ^2 ⩾4, N ⩾3
ROR	ROR= (A / B) / (C / D)	lower limit of 95% CI >1, N ⩾3
BCPNN	IC= $\log_2 \frac{P(AE,Drug)}{P(AE)P(Drug)}$<br><br>95% CI= E(IC) ± 2V(IC)^0.5	Lower limit of 95% CI ≥ 0
MGPS	EBGM= A(A+B+C+D)/(A+C)/(A+B)<br><br>95% CI= $e^{\ln(EBGM)\pm1.96(1/A+1/B+1/C+1/D)\text{^}0.5}$	Lower limit of 95% CI >1

AE: adverse event; PRR: proportional reporting ratio; ROR: reporting odd ratio; MGPS: multi-item Gamma Poisson shrinker; BCPNN: Bayesian confidence propagation neural network, IC: information component; EBGM = empirical Bayesian geometric mean; A: The number of a specific adverse event after each mRNA vaccine; B: The number of other adverse events after each mRNA vaccine; C: The number of a specific adverse event after all other vaccines; D: The number of other adverse events after all other vaccines; χ^2 = chi-square

Table S2. List of highly reported patient medical history in VAERS.

	Disease
1	Acute kidney injury
2	Anaemia
3	Aneurysm
4	Anxiety
5	Arrhythmia
6	Arthritis
7	Asthma
8	Benign prostatic hyperplasia
9	Cerebrovascular accident
10	Chronic kidney disease
11	Chronic obstructive pulmonary disease
12	Coronary artery disease
13	Crohn's disease
14	Deep vein thrombosis or Pulmonary embolism
15	Dementia
16	Depression
17	Fibromyalgia
18	Gastrooesophageal reflux disease
19	Heart failure
20	Hepatobiliary disorders
21	Hyperlipidemia
22	Hypertension
23	Hyperthyroidism
24	Hypothyroidism
25	Irritable bowel syndrome
26	Migraine
27	Multiple sclerosis
28	Myocardial infarction
29	Neoplasms benign, malignant and unspecified (incl cysts and polyps)
30	Osteoporosis
31	Polycystic ovaries
32	Pulmonary hypertension
33	Seizure
34	Sleep apnoea syndrome
35	Type 1 diabetes mellitus
36	Type 2 diabetes mellitus

Table S3. Clinical consequences of serious ICSRs after mRNA-based COVID-19 vaccination (n=555,033).

Category	Pfizer-BioNTech (BNT162b2)		Moderna (mRNA-1273)		P-value
	n	%	n	%	
ICSRs	266,369	100.00	288,664	100.00	
Non-serious ICSRs	238,102	89.39	265,433	91.95	p<.0000
Serious ICSRs	28,267	10.61	23,231	8.05	
Type of serious ICSRs†					
Death	3,924	1.47	3,840	1.33	p<.0000
Life-threatening	4,899	1.84	4,118	1.43	
Hospitalized/ prolonged	21,073	7.91	17,121	5.93	
Disability	5,605	2.10	4,140	1.43	
AEFIs	1,020,193	100.00	1,137,054	100.00	
Non-serious AEFIs	869,618	85.24	1,016,185	89.37	p<.0000
Serious AEFIs	150,575	14.76	120,869	10.63	

ICSR= individual case safety report; AEFI=adverse events after immunization

†: A report may meet more than one criterion for classification as type of seriousness

Table S4. Percentage changes between serious AEFIs and non-serious AEFIs by SOC after mRNA-based COVID-19 vaccination.

SOC	Pfizer-BioNTech (BNT162b2)					Moderna (mRNA-1273)				
	Non-serious AEFIs (n = 869,618)		Serious AEFIs (n = 150,575)		Change	Non-serious AEFIs (n = 1,016,185)		Serious AEFIs (n = 120,869)		Change
	n	%	n	%	%	n	%	n	%	%
Hepatobiliary disorders	289	0.03	642	0.43	1183	267	0.03	555	0.46	1648
Neoplasms benign, malignant and unspecified (incl cysts and polyps)	438	0.05	561	0.37	640	412	0.04	429	0.35	775
**Renal and urinary disorders**	**2,224**	**0.26**	**2,527**	**1.68**	**556**	**2,436**	**0.24**	**1,895**	**1.57**	**554**
Congenital, familial and genetic disorders	143	0.02	145	0.10	486	127	0.01	110	0.09	628
**Infections and infestations**	**22,146**	**2.55**	**16,898**	**11.22**	**341**	**17,988**	**1.77**	**11,406**	**9.44**	**433**
**Cardiac disorders**	**13,991**	**1.61**	**9,053**	**6.01**	**274**	**11,083**	**1.09**	**7,530**	**6.23**	**471**
Pregnancy, puerperium and perinatal conditions	1,052	0.12	675	0.45	271	737	0.07	366	0.30	318
Metabolism and nutrition disorders	5,993	0.69	3,456	2.30	233	7,297	0.72	2,710	2.24	212
Endocrine disorders	563	0.06	241	0.16	147	399	0.04	173	0.14	265
Respiratory, thoracic and mediastinal disorders	56,750	6.53	22,109	14.68	125	44,420	4.37	16,532	13.68	213
Vascular disorders	15,342	1.76	4,185	2.78	58	12,665	1.25	3,566	2.95	137
Immune system disorders	3,413	0.39	759	0.50	28	2,837	0.28	527	0.44	56
Psychiatric disorders	19,630	2.26	4,063	2.70	20	18,683	1.84	3,404	2.82	53
Ear and labyrinth disorders	15,604	1.79	2,399	1.59	-11	12,326	1.21	1,747	1.45	19
Eye disorders	15,851	1.82	2,355	1.56	-14	13,260	1.30	1,975	1.63	25
Nervous system disorders	153,620	17.67	21,790	14.47	-18	144,354	14.21	17,961	14.86	5
Blood and lymphatic system disorders	14,300	1.64	1,983	1.32	-20	11,179	1.10	1,586	1.31	19
Gastrointestinal disorders	77,817	8.95	10,455	6.94	-22	77,148	7.59	8,446	6.99	-8
General disorders and administration site conditions	273,959	31.50	32,699	21.72	-31	418,866	41.22	28,450	23.54	-43
Musculoskeletal and connective tissue disorders	90,784	10.44	9,463	6.28	-40	104,338	10.27	7,679	6.35	-38
Skin and subcutaneous tissue disorders	71,119	8.18	3,419	2.27	-72	106,550	10.49	3,356	2.78	-74
Reproductive system and breast disorders	14,590	1.68	698	0.46	-72	8,813	0.87	466	0.39	-56

AEFI: adverse events after immunization; SOC :system organ class.

Change (%) was calculated by subtracting proportion of non-serious AEFIs from proportion of serious AEFIs and dividing it by proportion of non-serious AEFIs.

Table S5. Signals detected from adverse events after administration of Pfizer-BioNTech (BNT162b2).

SOC	PT (n = 201)	Count	PRR	ROR	IC_{LB}	$EBGM_{LB}$
Blood and lymphatic system disorders	Lymphadenopathy	11,189	3.65	3.68	0.34	1.28
	Thrombocytopenia	354	3.04	3.04	0.13	1.16
	Lymph node pain	2,621	3.01	3.02	0.27	1.23
**Cardiac disorders**	Acute myocardial infarction	485	12.48	**12.49**	0.31	1.30
	Coronary artery occlusion	91	10.54	**10.54**	0.05	1.16
	Cardiac flutter	649	8.59	8.60	0.32	1.30
	Cardiomegaly	295	8.54	8.54	0.24	1.25
	Atrial flutter	122	8.07	8.08	0.10	1.18
	Supraventricular extrasystoles	130	7.53	7.53	0.10	1.18
	Cardiomyopathy	106	7.02	7.02	0.06	1.15
	Angina pectoris	542	6.98	6.98	0.28	1.27
	Cardiac disorder	416	6.42	6.43	0.25	1.25
	Myocardial infarction	660	6.00	6.00	0.29	1.27
	Extrasystoles	265	5.85	5.85	0.19	1.22
	Cardiac failure congestive	331	5.68	5.68	0.21	1.23
	Supraventricular tachycardia	229	5.58	5.59	0.17	1.20
	Cardiac failure	212	5.17	5.17	0.15	1.19
	Ventricular extrasystoles	442	5.12	5.12	0.23	1.24
	Sinus tachycardia	278	4.95	4.95	0.18	1.20
	Cardiac discomfort	147	4.86	4.87	0.08	1.15
	Cardiac arrest	523	4.66	4.66	0.24	1.23
	Atrial fibrillation	1,307	4.36	4.36	0.29	1.26
	Pericarditis	866	4.31	4.32	0.27	1.25
	Pericardial effusion	288	4.30	4.31	0.17	1.19
	Myocarditis	962	3.98	3.98	0.26	1.24
	Cardio-respiratory arrest	145	3.95	3.95	0.05	1.13
	Bradycardia	353	3.89	3.90	0.18	1.19
	Palpitations	7,137	3.84	3.87	0.34	1.28
	Arrhythmia	512	3.71	3.71	0.20	1.21
	Tachycardia	2,908	3.57	3.58	0.30	1.26
Ear and labyrinth disorders	Ear discomfort	1,372	4.30	4.30	0.29	1.26
	Tinnitus	6,935	4.23	4.25	0.35	1.29
	Vertigo	3,796	2.63	2.64	0.25	1.21
	Hypoacusis	693	2.57	2.57	0.16	1.16
	Hyperacusis	334	2.34	2.35	0.07	1.11
	Deafness	706	2.32	2.32	0.14	1.14
	Ear pain	1,852	2.05	2.05	0.17	1.15
Eye disorders	Eye haemorrhage	146	4.83	4.83	0.08	1.15
	Blepharospasm	308	3.48	3.48	0.14	1.17
	Vitreous floaters	289	3.27	3.27	0.12	1.16
	Asthenopia	190	3.04	3.04	0.06	1.12
	Ocular discomfort	405	2.76	2.76	0.13	1.15
Gastrointestinal disorders	Gastrointestinal haemorrhage	181	8.39	8.39	0.17	1.21
	Tongue pruritus	286	5.30	5.30	0.19	1.21
	Paraesthesia oral	3,500	3.68	3.69	0.31	1.26
	Hypoaesthesia oral	2,491	3.68	3.68	0.30	1.26
	Tongue discomfort	292	3.38	3.38	0.13	1.16

SOC	PT (n = 201)	Count	PRR	ROR	IC_{LB}	$EBGM_{LB}$
	Haematochezia	263	2.97	2.97	0.10	1.14
	Oral pruritus	275	2.90	2.90	0.10	1.14
	Tongue disorder	259	2.86	2.86	0.09	1.13
	Swollen tongue	2,282	2.68	2.69	0.24	1.20
	Abdominal pain lower	375	2.56	2.56	0.11	1.13
	Oral discomfort	387	2.36	2.36	0.09	1.12
	Gastrooesophageal reflux disease	368	2.30	2.30	0.08	1.11
	Dyspepsia	810	2.23	2.23	0.14	1.14
	Dry mouth	995	2.14	2.15	0.14	1.14
	Abdominal distension	781	2.10	2.10	0.12	1.13
	Abdominal pain	3,210	2.08	2.09	0.19	1.16
	Mouth swelling	453	2.06	2.06	0.07	1.10
	Dysphagia	2,453	2.05	2.05	0.18	1.15
General disorders and administration site conditions	Disease recurrence	189	87.57	**87.58**	0.24	1.28
	Vaccination site pruritus	596	16.24	**16.25**	0.34	1.33
	Drug ineffective	754	15.19	**15.20**	0.36	1.33
	Facial discomfort	133	6.85	6.85	0.10	1.17
	Illness	1,426	6.67	6.68	0.35	1.31
	Death	4,033	5.82	5.84	0.38	1.32
	General physical health deterioration	350	5.79	5.79	0.22	1.23
	Vaccination site mass	381	5.19	5.19	0.22	1.23
	Vaccination site erythema	1,073	5.07	5.08	0.30	1.27
	Vaccination site pain	5,135	4.76	4.78	0.36	1.30
	Therapeutic response unexpected	187	3.94	3.94	0.09	1.15
	Chest pain	10,518	3.41	3.44	0.33	1.27
	Chest discomfort	7,688	3.38	3.40	0.32	1.26
	Feeling jittery	319	2.74	2.74	0.10	1.14
	Condition aggravated	6,965	2.59	2.60	0.27	1.22
	Sensation of foreign body	561	2.18	2.18	0.11	1.12
	Vaccination site rash	380	2.10	2.10	0.06	1.10
Immune system disorders	Immunodeficiency	400	5.79	5.79	0.24	1.24
	Anaphylactic reaction	966	2.12	2.12	0.14	1.14
Infections and infestations	Asymptomatic COVID-19	588	136.21	**136.29**	0.38	1.36
	COVID-19 pneumonia	1,903	58.78	**58.89**	0.45	1.40
	Vaccine breakthrough infection	1,584	45.87	**45.94**	0.43	1.39
	COVID-19	18,765	44.82	**45.64**	0.51	1.43
	Appendicitis	406	20.90	**20.91**	0.32	1.31
	Suspected COVID-19	209	10.76	**10.76**	0.21	1.24
	Ear infection	195	3.93	3.93	0.10	1.15
	Sinusitis	466	2.57	2.57	0.13	1.15
	Urinary tract infection	706	2.35	2.35	0.14	1.15
	Sepsis	536	2.24	2.24	0.11	1.13
	Pneumonia	1,446	2.21	2.21	0.17	1.16
Metabolism and nutrition disorders	Hyperglycaemia	148	5.71	5.71	0.10	1.17
	Hypokalaemia	144	4.77	4.77	0.08	1.15
	Hyponatraemia	333	4.68	4.68	0.19	1.21
	Hypophagia	353	2.77	2.77	0.12	1.14

SOC	PT (n = 201)	Count	PRR	ROR	IC_{LB}	$EBGM_{LB}$
Musculoskeletal and connective tissue disorders	Costochondritis	119	13.78	**13.79**	0.12	1.20
	Axillary mass	461	2.43	2.43	0.12	1.13
	Muscle twitching	1,164	2.07	2.08	0.15	1.14
Nervous system disorders	Anosmia	2,052	24.38	**24.42**	0.43	1.38
	Cerebral infarction	171	13.20	**13.21**	0.19	1.23
	Ageusia	2,415	12.71	**12.74**	0.42	1.36
	Taste disorder	943	7.80	7.81	0.34	1.31
	Parosmia	688	7.24	7.25	0.31	1.29
	Electric shock sensation	390	7.23	7.23	0.26	1.26
	Bell's palsy	1,659	6.99	7.00	0.36	1.32
	Ischaemic stroke	192	5.93	5.93	0.15	1.19
	Cerebrovascular accident	1,306	4.76	4.77	0.31	1.27
	Cerebral haemorrhage	189	4.38	4.38	0.11	1.16
	Dysgeusia	2,273	3.71	3.71	0.30	1.26
	Movement disorder	720	3.40	3.41	0.22	1.21
	Transient ischaemic attack	306	3.02	3.02	0.12	1.15
	Head discomfort	1,457	2.97	2.98	0.24	1.21
	Dizziness postural	322	2.71	2.71	0.10	1.13
	Migraine	3,990	2.70	2.71	0.26	1.22
	Hemiparesis	403	2.63	2.63	0.12	1.14
	Aphasia	763	2.56	2.56	0.17	1.17
	Cognitive disorder	390	2.54	2.55	0.11	1.13
	Unresponsive to stimuli	1,811	2.45	2.45	0.21	1.18
	Neurological symptom	311	2.36	2.36	0.07	1.11
	Memory impairment	792	2.24	2.24	0.14	1.14
	Dysarthria	720	2.06	2.06	0.11	1.12
Pregnancy, puerperium and perinatal conditions	Abortion spontaneous	689	4.20	4.20	0.25	1.23
Psychiatric disorders	Panic attack	635	3.98	3.98	0.23	1.22
	Anxiety	3,870	3.32	3.33	0.30	1.25
	Mental status changes	623	3.10	3.10	0.19	1.19
	Nervousness	1,004	2.45	2.45	0.18	1.17
	Fear	317	2.26	2.26	0.06	1.10
	Depression	543	2.11	2.11	0.10	1.12
Renal and urinary disorders	Chronic kidney disease	137	10.58	**10.58**	0.14	1.20
	Acute kidney injury	1,052	10.15	**10.16**	0.36	1.33
	Haematuria	181	3.65	3.65	0.08	1.14
	Renal failure	184	3.55	3.55	0.08	1.14
	Dysuria	293	2.30	2.30	0.06	1.10
Reproductive system and breast disorders	Oligomenorrhoea	328	50.66	**50.67**	0.31	1.32
	Premenstrual syndrome	88	40.77	**40.78**	0.09	1.19
	Postmenopausal haemorrhage	288	33.36	**33.37**	0.29	1.30
	Menstruation delayed	654	30.30	**30.32**	0.37	1.35
	Dysmenorrhoea	1,110	28.57	**28.60**	0.40	1.37
	Polymenorrhoea	408	27.00	**27.02**	0.32	1.32
	Hypomenorrhoea	106	24.56	**24.56**	0.12	1.20
	Intermenstrual bleeding	829	24.01	**24.02**	0.38	1.35
	Heavy menstrual bleeding	2,604	21.17	**21.22**	0.44	1.39
	Menstruation irregular	1,935	20.38	**20.41**	0.43	1.38
	Menstrual disorder	1,464	17.85	**17.87**	0.41	1.36

SOC	PT (n = 201)	Count	PRR	ROR	IC_{LB}	$EBGM_{LB}$
	Breast mass	218	10.10	**10.10**	0.21	1.24
	Vaginal haemorrhage	936	10.09	**10.09**	0.35	1.32
	Breast tenderness	268	6.21	6.21	0.20	1.22
	Amenorrhoea	374	4.81	4.81	0.21	1.22
	Breast swelling	388	3.52	3.53	0.17	1.18
	Pelvic pain	198	3.06	3.06	0.06	1.12
	Breast pain	994	2.69	2.69	0.20	1.18
Respiratory, thoracic and mediastinal disorders	Acute respiratory failure	1,120	34.59	**34.63**	0.41	1.37
	Lung opacity	786	33.11	**33.13**	0.38	1.36
	Lung consolidation	125	14.48	**14.48**	0.13	1.21
	Hypoxia	1,358	13.68	**13.69**	0.39	1.35
	Pulmonary embolism	1,421	11.97	**11.99**	0.39	1.35
	Atelectasis	277	11.67	**11.67**	0.25	1.27
	Throat clearing	201	9.31	9.31	0.19	1.23
	Lung infiltration	423	8.52	8.52	0.28	1.28
	Pulmonary mass	159	7.37	7.37	0.14	1.19
	Pulmonary thrombosis	269	6.92	6.93	0.21	1.23
	Pleural effusion	420	6.28	6.28	0.25	1.25
	Chronic obstructive pulmonary disease	261	5.76	5.76	0.19	1.22
	Oropharyngeal discomfort	692	5.62	5.63	0.28	1.27
	Pharyngeal paraesthesia	522	5.15	5.15	0.25	1.24
	Dyspnoea exertional	774	4.91	4.92	0.28	1.26
	Pulmonary oedema	245	4.73	4.73	0.16	1.19
	Lung disorder	236	4.21	4.21	0.14	1.18
	Epistaxis	1,013	3.91	3.91	0.26	1.24
	Pharyngeal hypoaesthesia	259	3.87	3.87	0.14	1.17
	Hyperventilation	359	3.78	3.78	0.17	1.19
	Paranasal sinus discomfort	439	3.77	3.77	0.19	1.20
	Haemoptysis	192	3.71	3.71	0.09	1.15
	Tachypnoea	283	3.64	3.64	0.14	1.17
	Respiratory distress	308	3.40	3.40	0.14	1.17
	Pulmonary pain	247	3.18	3.18	0.10	1.14
	Pulmonary congestion	233	3.18	3.18	0.09	1.14
	Nasal congestion	2,714	3.14	3.15	0.28	1.23
	Pharyngeal swelling	2,145	3.14	3.14	0.27	1.23
	Respiratory failure	365	3.13	3.13	0.14	1.16
	Cough	11,386	3.00	3.02	0.31	1.25
	Sinus congestion	614	2.99	3.00	0.18	1.18
	Respiratory tract congestion	2,132	2.91	2.92	0.25	1.22
	Upper-airway cough syndrome	317	2.88	2.88	0.11	1.14
	Rhinorrhoea	3,438	2.81	2.82	0.26	1.22
	Dyspnoea	19,740	2.72	2.75	0.29	1.24
	Dry throat	439	2.71	2.71	0.13	1.15
	Throat tightness	3,399	2.56	2.56	0.24	1.20
	Throat irritation	3,123	2.50	2.51	0.24	1.20
	Sneezing	911	2.37	2.37	0.16	1.16
	Productive cough	917	2.22	2.23	0.15	1.15
	Asthma	689	2.11	2.11	0.12	1.12
	Oropharyngeal pain	5,642	2.08	2.09	0.21	1.17

SOC	PT (n = 201)	Count	PRR	ROR	IC_{LB}	$EBGM_{LB}$
Skin and subcutaneous tissue disorders	Alopecia	564	2.18	2.18	0.11	1.12
Vascular disorders	Superficial vein thrombosis	124	57.45	**57.46**	0.16	1.23
	Deep vein thrombosis	1,012	13.02	**13.04**	0.37	1.34
	Thrombosis	1,582	8.24	8.25	0.37	1.33
	Hypertension	3,051	3.83	3.84	0.31	1.27
	Blood pressure fluctuation	177	3.73	3.73	0.08	1.14
	Hot flush	1,731	2.83	2.84	0.24	1.21
	Flushing	4,370	2.61	2.62	0.25	1.21
	Hypotension	1,746	2.01	2.01	0.16	1.14

SOC= system organ class; PT= preferred term; PRR = proportional reporting ratio; ROR = reporting odd ratio; IC_{LB} = lower bound of information component 95% confidence interval; $EBGM_{LB}$ = lower bound of empirical bayesian geometric mean 95% confidence interval

Table S6. Signals detected in adverse events after administration of Moderna (mRNA-1273).

SOC	PT (n = 110)	Count	PRR	ROR	IC_{LB}	$EBGM_{LB}$
Blood and lymphatic system disorders	Lymphadenopathy	8,683	2.54	2.56	0.24	1.20
	Lymph node pain	2,035	2.10	2.10	0.16	1.14
Cardiac disorders	Acute myocardial infarction	366	8.45	8.45	0.22	1.23
	Cardiomegaly	236	6.13	6.13	0.14	1.18
	Cardiac flutter	478	5.68	5.68	0.22	1.22
	Myocardial infarction	582	4.74	4.75	0.21	1.21
	Cardiac failure congestive	296	4.56	4.56	0.14	1.17
	Supraventricular tachycardia	194	4.24	4.25	0.08	1.14
	Cardiac failure	181	3.96	3.96	0.06	1.12
	Extrasystoles	194	3.84	3.84	0.06	1.13
	Cardiac disorder	275	3.81	3.81	0.11	1.15
	Ventricular extrasystoles	366	3.80	3.80	0.14	1.17
	Atrial fibrillation	1,232	3.68	3.69	0.23	1.21
	Sinus tachycardia	223	3.57	3.57	0.07	1.13
	Angina pectoris	305	3.52	3.52	0.11	1.15
	Cardiac arrest	433	3.46	3.46	0.15	1.16
	Pericardial effusion	241	3.23	3.23	0.07	1.12
	Myocarditis	761	2.82	2.83	0.16	1.16
	Palpitations	5,692	2.75	2.76	0.25	1.20
	Pericarditis	612	2.74	2.74	0.14	1.15
	Tachycardia	2,451	2.70	2.71	0.22	1.19
Ear and labyrinth disorders	Tinnitus	5,295	2.90	2.91	0.25	1.21
	Ear discomfort	994	2.79	2.79	0.18	1.17
	Vertigo	3,226	2.01	2.01	0.16	1.14
Eye disorders	Ocular discomfort	369	2.26	2.26	0.05	1.09
Gastrointestinal disorders	Paraesthesia oral	2,623	2.47	2.48	0.20	1.17
	Hypoaesthesia oral	1,864	2.47	2.47	0.19	1.17
General disorders and administration site conditions	Vaccination site pruritus	2,929	71.62	**71.80**	0.42	1.36
	Vaccination site urticaria	226	46.97	**46.98**	0.21	1.25
	Vaccination site erythema	4,343	18.42	**18.49**	0.41	1.35
	Vaccination site rash	1,913	9.47	9.48	0.35	1.30
	Vaccination site mass	673	8.23	8.23	0.28	1.26
	Illness	1,903	7.99	8.00	0.34	1.29
	Vaccination site movement impairment	229	7.93	7.93	0.16	1.20
	Facial discomfort	132	6.10	6.10	0.05	1.13
	Vaccination site pain	7,138	5.93	5.97	0.36	1.30
	Vaccination site induration	969	5.52	5.52	0.27	1.25
	Vaccination site discolouration	232	5.36	5.36	0.13	1.17
	Death	3,685	4.77	4.78	0.32	1.27
	General physical health deterioration	283	4.20	4.20	0.13	1.16
	Vaccination site reaction	916	4.05	4.05	0.23	1.21
	Therapeutic response unexpected	204	3.85	3.86	0.07	1.13
	Vaccination site warmth	2,454	3.57	3.57	0.26	1.23
	Vaccination site swelling	3,387	3.27	3.27	0.26	1.22
	Chest pain	7,751	2.26	2.27	0.21	1.17

SOC	PT (n = 110)	Count	PRR	ROR	IC_{LB}	$EBGM_{LB}$
	Chest discomfort	5,624	2.22	2.22	0.20	1.17
Infections and infestations	Asymptomatic COVID-19	320	66.51	**66.53**	0.26	1.28
	COVID-19 pneumonia	1,123	31.12	**31.15**	0.36	1.33
	COVID-19	11,848	25.39	**25.64**	0.44	1.37
	Vaccine breakthrough infection	925	24.03	**24.05**	0.34	1.32
	Post-acute COVID-19 syndrome	215	12.77	**12.77**	0.18	1.22
	Appendicitis	206	9.51	9.52	0.16	1.20
	Urinary tract infection	775	2.32	2.32	0.12	1.13
	Sinusitis	448	2.22	2.22	0.07	1.10
Nervous system disorders	Anosmia	1,400	14.92	**14.94**	0.35	1.32
	Ageusia	1,877	8.87	8.88	0.34	1.30
	Taste disorder	938	6.96	6.97	0.29	1.26
	Bell's palsy	1,380	5.22	5.22	0.28	1.25
	Electric shock sensation	301	5.01	5.01	0.15	1.18
	Parosmia	521	4.92	4.92	0.21	1.21
	Cerebrovascular accident	1,267	4.15	4.15	0.25	1.23
	Movement disorder	687	2.91	2.92	0.16	1.16
	Dysgeusia	1,649	2.41	2.42	0.18	1.16
	Hypokinesia	414	2.33	2.33	0.07	1.10
	Migraine	3,612	2.19	2.20	0.19	1.16
	Aphasia	727	2.19	2.19	0.11	1.12
	Head discomfort	1,148	2.10	2.10	0.13	1.12
Pregnancy, puerperium and perinatal conditions	Abortion spontaneous	420	2.30	2.30	0.07	1.10
Psychiatric disorders	Panic attack	459	2.58	2.58	0.10	1.13
	Mental status changes	528	2.36	2.36	0.10	1.12
	Anxiety	2,882	2.22	2.22	0.18	1.16
Renal and urinary disorders	Acute kidney injury	643	5.57	5.57	0.24	1.23
Reproductive system and breast disorders	Oligomenorrhoea	199	27.57	**27.58**	0.19	1.23
	Postmenopausal haemorrhage	184	19.12	**19.13**	0.17	1.22
	Menstruation delayed	385	16.00	**16.01**	0.26	1.26
	Dysmenorrhoea	624	14.41	**14.42**	0.30	1.28
	Polymenorrhoea	218	12.95	**12.95**	0.18	1.22
	Intermenstrual bleeding	465	12.08	**12.09**	0.26	1.26
	Heavy menstrual bleeding	1,487	10.84	**10.86**	0.34	1.30
	Menstruation irregular	1,097	10.36	**10.37**	0.32	1.29
	Menstrual disorder	827	9.05	9.05	0.30	1.28
	Breast mass	145	6.03	6.03	0.07	1.14
	Vaginal haemorrhage	514	4.97	4.97	0.21	1.21
	Breast tenderness	211	4.39	4.39	0.09	1.15
Respiratory, thoracic and mediastinal disorders	Acute respiratory failure	727	20.15	**20.16**	0.32	1.30
	Lung opacity	489	18.48	**18.49**	0.29	1.28
	Pulmonary embolism	1,308	9.89	9.90	0.33	1.30
	Hypoxia	1,049	9.48	9.49	0.32	1.29
	Atelectasis	198	7.48	7.48	0.13	1.18
	Throat clearing	157	6.53	6.53	0.09	1.15
	Lung infiltration	332	6.00	6.00	0.18	1.20
	Pulmonary thrombosis	250	5.77	5.77	0.15	1.18
	Oropharyngeal discomfort	607	4.43	4.43	0.21	1.21

SOC	PT (n = 110)	Count	PRR	ROR	IC_{LB}	$EBGM_{LB}$
	Chronic obstructive pulmonary disease	219	4.34	4.34	0.10	1.15
	Pleural effusion	322	4.32	4.32	0.15	1.17
	Pulmonary oedema	228	3.95	3.95	0.09	1.14
	Pharyngeal paraesthesia	355	3.14	3.14	0.11	1.14
	Dyspnoea exertional	537	3.06	3.06	0.15	1.16
	Tachypnoea	253	2.92	2.92	0.06	1.11
	Epistaxis	829	2.87	2.87	0.17	1.17
	Paranasal sinus discomfort	329	2.53	2.53	0.06	1.11
	Pharyngeal swelling	1,748	2.29	2.29	0.17	1.15
	Dyspnoea	16,455	2.03	2.05	0.20	1.16
	Nasal congestion	1,957	2.03	2.04	0.15	1.13
Skin and subcutaneous tissue disorders	Mechanical urticaria	268	15.92	**15.92**	0.21	1.24
	Skin swelling	414	2.65	2.65	0.10	1.12
Vascular disorders	Deep vein thrombosis	934	10.79	**10.79**	0.32	1.29
	Thrombosis	1,198	5.60	5.60	0.28	1.25
	Hypertension	2,606	2.94	2.94	0.23	1.20
	Hot flush	1,512	2.22	2.22	0.15	1.14

SOC= system organ class; PT= preferred term; PRR = proportional reporting ratio; ROR = reporting odd ratio; IC_{LB} = lower bound of information component 95% confidence interval; $EBGM_{LB}$ = lower bound of empirical bayesian geometric mean 95% confidence interval

Table S7. Signals detected from serious adverse events after administration of Pfizer-BioNTech (BNT162b2).

SOC	PT (n = 28)	Count	PRR	ROR	IC_{LB}	$EBGM_{LB}$
Cardiac disorders	Acute myocardial infarction	467	10.72	**10.75**	0.15	1.17
	Myocardial infarction	526	3.83	3.84	0.09	1.12
	Atrial fibrillation	814	3.52	3.54	0.12	1.13
	Cardiac arrest	510	3.17	3.18	0.07	1.10
General disorders and administration site conditions	Death	4,021	3.79	3.86	0.21	1.17
	Unevaluable event	943	3.52	3.53	0.13	1.13
	Condition aggravated	2,200	2.52	2.54	0.13	1.12
	Chest pain	2,439	2.09	2.11	0.11	1.10
Infections and infestations	Vaccine breakthrough infection	1,155	114.93	**115.81**	0.25	1.23
	COVID-19	8,628	37.33	**39.54**	0.32	1.27
	COVID-19 pneumonia	1,831	39.04	**39.51**	0.27	1.24
	Asymptomatic COVID-19	256	38.21	**38.28**	0.11	1.16
	Appendicitis	354	17.61	**17.65**	0.14	1.16
Nervous system disorders	Cerebrovascular accident	1,139	3.47	3.49	0.14	1.14
Renal and urinary disorders	Acute kidney injury	1,001	7.47	7.51	0.19	1.18
Respiratory, thoracic and mediastinal disorders	Lung opacity	748	22.33	**22.44**	0.21	1.20
	Acute respiratory failure	1,100	21.89	**22.05**	0.24	1.22
	Chronic obstructive pulmonary disease	217	10.80	**10.81**	0.06	1.12
	Atelectasis	233	9.94	9.95	0.07	1.12
	Hypoxia	1,280	9.10	9.17	0.22	1.20
	Pulmonary embolism	1,237	8.21	8.27	0.21	1.19
	Lung infiltration	408	6.09	6.10	0.11	1.14
	Pleural effusion	359	4.87	4.88	0.08	1.12
	Cough	3,093	4.33	4.40	0.21	1.18
	Dyspnoea exertional	468	4.23	4.24	0.09	1.12
	Dyspnoea	6,042	2.47	2.53	0.16	1.13
Vascular disorders	Deep vein thrombosis	603	6.92	6.95	0.15	1.16
	Thrombosis	645	3.93	3.94	0.11	1.13

SOC= system organ class; PT= preferred term; PRR = proportional reporting ratio; ROR = reporting odd ratio; IC_{LB} = lower bound of information component 95% confidence interval; $EBGM_{LB}$ = lower bound of empirical bayesian geometric mean 95% confidence interval

Table S8. Signals detected from serious adverse events after administration of Moderna (mRNA-1273).

SOC	PT (n=37)	Count	PRR	ROR	IC_{LB}	$EBGM_{LB}$
Cardiac disorders	Acute myocardial infarction	358	10.24	**10.27**	0.19	1.21
	Cardiomegaly	190	7.07	7.08	0.09	1.15
	Cardiac failure congestive	265	4.93	4.94	0.10	1.15
	Myocardial infarction	500	4.54	4.55	0.16	1.17
	Atrial fibrillation	703	3.79	3.81	0.17	1.17
	Cardiac arrest	422	3.27	3.28	0.11	1.13
	Myocarditis	547	2.14	2.15	0.06	1.09
General disorders and administration site conditions	Death	3,673	4.31	4.41	0.27	1.23
	General physical health deterioration	257	3.98	3.99	0.08	1.13
	Unevaluable event	703	3.27	3.28	0.15	1.16
	Condition aggravated	1,727	2.46	2.48	0.16	1.14
	Chest pain	1,894	2.02	2.04	0.12	1.12
Infections and infestations	Vaccine breakthrough infection	667	82.69	**83.14**	0.29	1.28
	COVID-19	5,515	29.72	**31.10**	0.38	1.32
	Asymptomatic COVID-19	159	29.57	**29.60**	0.11	1.18
	COVID-19 pneumonia	1,076	28.58	**28.83**	0.31	1.29
	Appendicitis	167	10.35	**10.36**	0.09	1.15
	Urinary tract infection	344	3.20	3.20	0.08	1.12
	Pneumonia	950	2.10	2.11	0.10	1.10
Nervous system disorders	Cerebrovascular accident	1,091	4.14	4.17	0.21	1.20
	Unresponsive to stimuli	491	2.34	2.35	0.07	1.10
Psychiatric disorders	Mental status changes	439	2.44	2.44	0.06	1.10
Renal and urinary disorders	Acute kidney injury	620	5.76	5.79	0.21	1.20
Respiratory, thoracic and mediastinal disorders	Acute respiratory failure	724	17.95	**18.05**	0.28	1.26
	Lung opacity	468	17.40	**17.47**	0.24	1.24
	Chronic obstructive pulmonary disease	171	10.60	**10.61**	0.09	1.16
	Pulmonary embolism	1,159	9.58	9.66	0.28	1.26
	Atelectasis	174	9.24	9.26	0.09	1.15
	Hypoxia	979	8.67	8.73	0.27	1.25
	Lung infiltration	318	5.91	5.93	0.14	1.17
	Pulmonary thrombosis	232	5.39	5.40	0.09	1.14
	Pleural effusion	281	4.75	4.76	0.11	1.15
	Dyspnoea exertional	329	3.71	3.72	0.10	1.13
	Cough	1,946	3.40	3.44	0.22	1.19
	Dyspnoea	4,710	2.40	2.46	0.19	1.16
Vascular disorders	Deep vein thrombosis	544	7.78	7.81	0.22	1.22
	Thrombosis	510	3.87	3.88	0.15	1.16

SOC= system organ class; PT= preferred term; PRR = proportional reporting ratio; ROR = reporting odd ratio; IC_{LB} = lower bound of information component 95% confidence interval; $EBGM_{LB}$ = lower bound of empirical bayesian geometric mean 95% confidence interval

Table S9. Area under the receiver operating characteristic curve (AUROC) with various numbers of selected features in major signals of AEFIs.

<table>
<tr><th></th><th>feature selection 20</th><th>feature selection 10</th><th>feature selection 7</th><th>feature selection 5</th><th>feature selection 3</th></tr>
<tr><td>AF</td>
<td>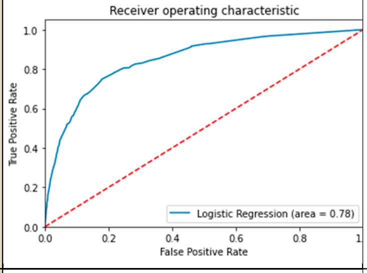
</td>
<td>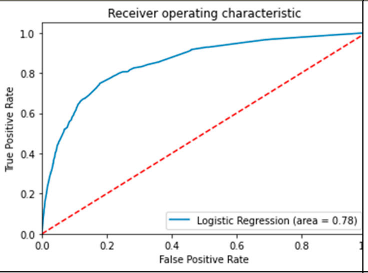
</td>
<td>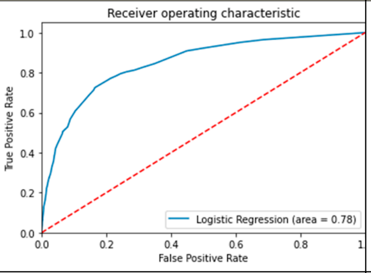
</td>
<td>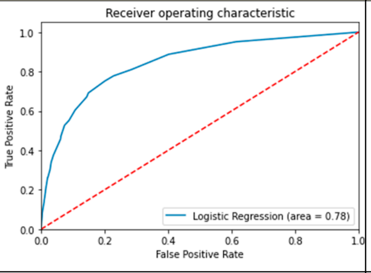
</td>
<td>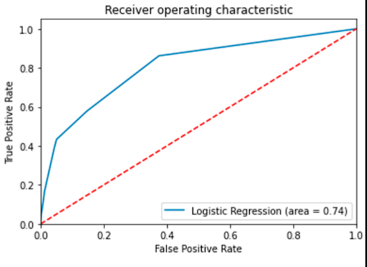
</td></tr>
<tr><td>AKI</td>
<td>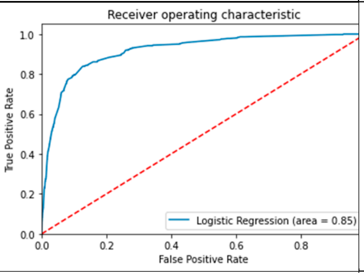
</td>
<td>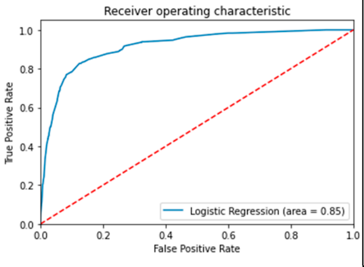
</td>
<td>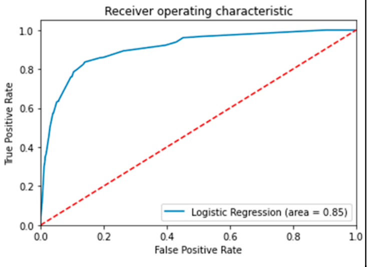
</td>
<td>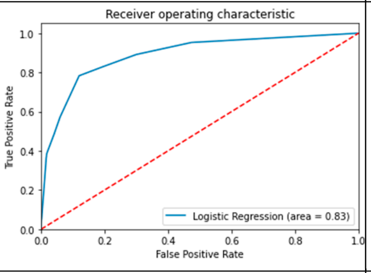
</td>
<td>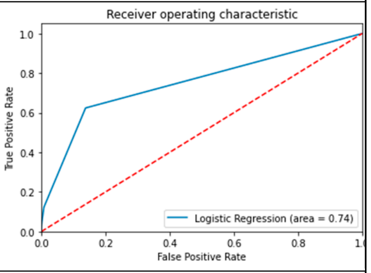
</td></tr>
<tr><td>CVA</td>
<td>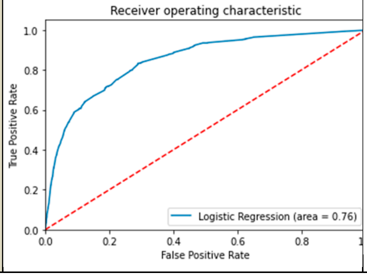
</td>
<td>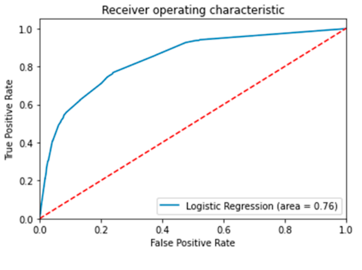
</td>
<td>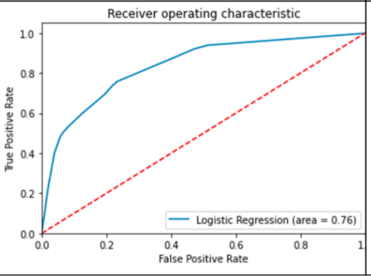
</td>
<td>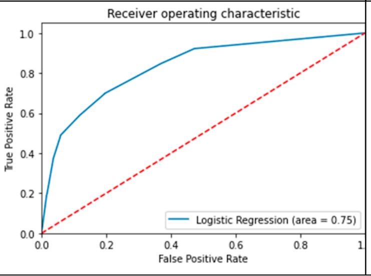
</td>
<td>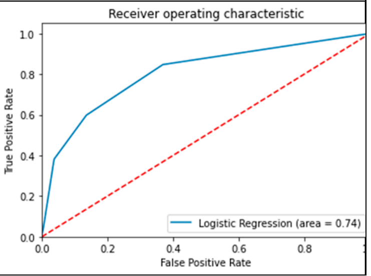
</td></tr>
</table>

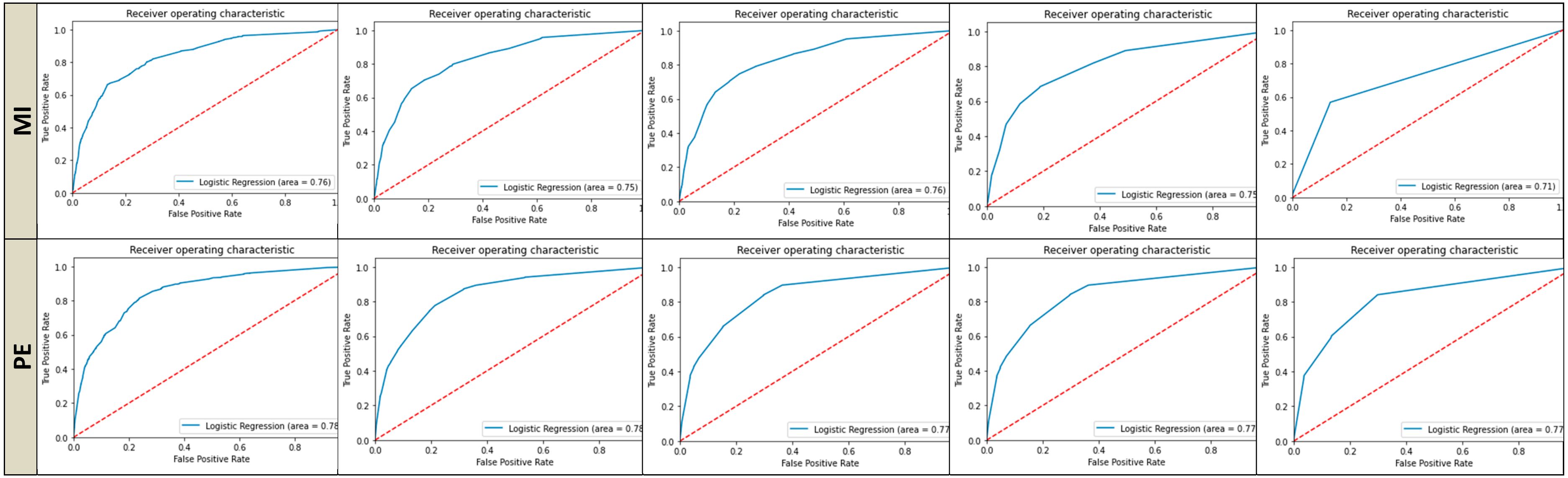
MI
PE
Receiver operating characteristic
True Positive Rate
False Positive Rate
Logistic Regression (area = 0.76)
Logistic Regression (area = 0.75)
Logistic Regression (area = 0.76)
Logistic Regression (area = 0.75
Logistic Regression (area = 0.71)
Logistic Regression (area = 0.78
Logistic Regression (area = 0.78
Logistic Regression (area = 0.77
Logistic Regression (area = 0.77
Logistic Regression (area = 0.77

(days).
